# Adult Male with Traumatic Eye Pain and Swelling

**DOI:** 10.5811/cpcem.2018.2.36857

**Published:** 2018-04-05

**Authors:** Marc Leshner, Ryan Gibbons, Thomas Costantino

**Affiliations:** Lewis Katz School of Medicine at Temple University, Department of Emergency Medicine, Philadelphia, Pennsylvania

## Case Presentation

**Image 1:**
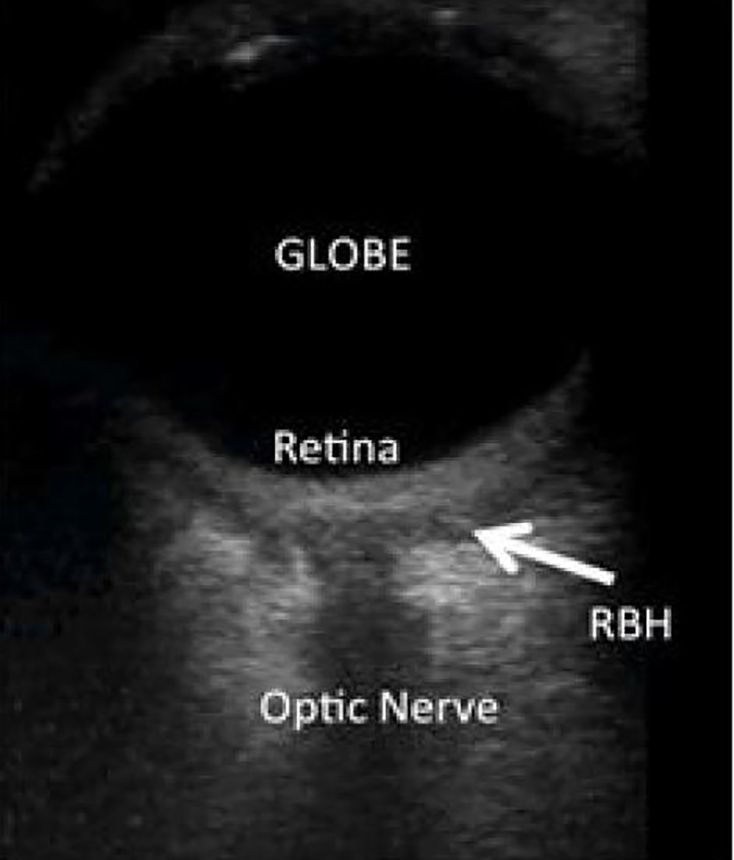
Point-of-care ultrasound, transverse view, of the left orbit using a linear probe demonstrating a RBH (arrow). *RBH*, retrobulbar hematoma.

**Image 2:**
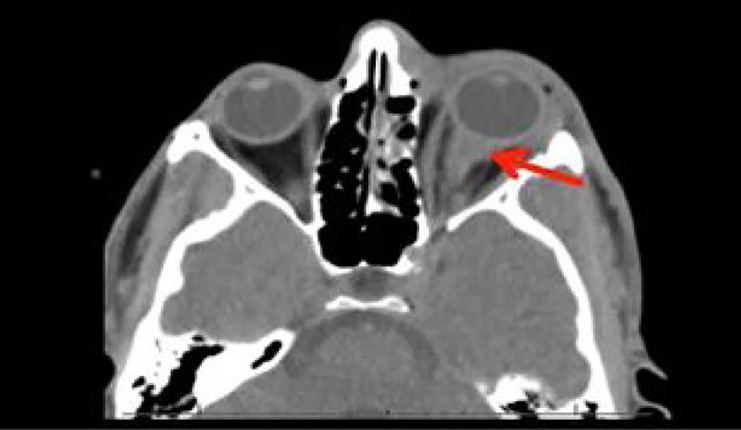
Computed tomography maxillofacial without contrast demonstrating an intraconal hematoma (red arrow), thickening of the optic nerve with proptosis

A 28-year-old male, presented to the emergency department following assault with a fist to the left eye. The patient complained of pain and blurry vision but denied diplopia. Physical examination was significant for left-sided periorbital ecchymosis with a subconjunctival hemorrhage. Both pupils were equal and reactive to light. Visual acuity was 20/30 in the right eye and 20/20 in the left. Eye and intraocular pressures measured 13 and 17 respectively. No proptosis was observed. Point-of-care ocular ultrasound was performed followed by computed tomography (CT) maxillofacial without contrast (Images 1 and 2 respectively).

## Diagnosis

The patient was found to have a left retrobulbar hematoma (RBH) that was diagnosed immediately by performing point-of-care ocular ultrasound. This was confirmed with CT imaging. Image 1 demonstrates a left orbital ultrasound with hypoechoic material within the retrobulbar space suggestive of a RBH.^1^ Image 2 illustrates an intraconal hematoma and thickening of the optic nerve complex with proptosis, characteristic of a RBH.^2^

RBH is a rapidly progressing ocular emergency that can lead to permanent vision loss.^3^ The accumulation of blood in the retrobulbar space can lead to an orbital compartment syndrome causing compressive ischemia to the optic nerve that can lead to blindness if prompt lateral canthotomy is not performed.^4^ Diagnosis of RBH is challenging, but clinical clues include severe pain, proptosis, vision loss and an afferent pupillary defect.^3^ Although the condition has not been studied clinically, animal and cadaver research suggests ultrasound has a high sensitivity and specificity for the diagnosis of RBH.^4^,^5^

In the case described above, the clinical history was concerning for RBH, and an immediate point-of-care ocular ultrasound confirmed our suspicion. Notably, the patient did not demonstrate evidence of globe rupture, which would be a contraindication of ocular ultrasound. An emergent canthotomy was deferred for ophthalmology given the normal intraocular pressure in the left eye. This case highlights that in select patients with suspected RBH, point-of-care ocular ultrasound can expedite the diagnosis without the delay of CT, and thus timely ocular decompression can be performed to prevent vision loss.

**CPC-EM Capsule**

What do we already know about this clinical entity?

Retrobulbar hematoma (RBH) is a rapidly progressing ocular emergency that is often seen in the setting of ocular trauma. It can lead to permanent vision loss if prompt lateral canthotomy is not performed.

What makes this presentation of disease reportable?

While RBH is a rare clinical entity, emergency physicians need to be comfortable with diagnosing and treating this condition.

What is the major learning point?

Point-of-care ocular ultrasound is a quick and accurate imaging modality to diagnose RBH.

How might this improve emergency medicine practice?

In cases where it is suspected, RBH can be diagnosed via point-of-care ocular ultrasound eliminating the need for unnecessary and time-consuming computed tomography, thus, allowing for more rapid ocular decompression.

Documented patient informed consent and/or Institutional Review Board approval has been obtained and filed for publication of this case report.

## References

[1] Blaivas M (2000). Bedside emergency department ultrasonography in the evaluation of ocular pathology. Acad Emerg Med.

[2] Cobb SR, Yeakley JW, Lee KF (1985). Computed tomographic evaluation of ocular trauma. Comput Radiol.

[3] Bailey WK, Kuo PC, Evans LS (1993). Diagnosis and treatment of retrobulbar hemorrhage. J Oral Maxillofac Surg.

[4] Gerbino G, Ramieri GA, Nasi A (2005). Diagnosis and treatment of retrobulbar haematomas following blunt orbital trauma: a description of eight cases. Int J Oral Maxillofac Surg.

[5] Estevez A, Deutch J, Sturmann K (2000). Ultrasonographic evaluation of retrobulbar hematoma in bovine orbits. Ann Emerg Med.

